# *Polymorphus Minutus* Affects Antitoxic Responses of *Gammarus Roeseli* Exposed to Cadmium

**DOI:** 10.1371/journal.pone.0041475

**Published:** 2012-07-20

**Authors:** Eric Gismondi, Jean-Nicolas Beisel, Carole Cossu-Leguille

**Affiliations:** Laboratoire des Interactions Ecotoxicologie Biodiversité Ecosystèmes (LIEBE), CNRS UMR 7146, Université de Lorraine, Metz, France; University of Osnabrueck, Germany

## Abstract

The acanthocephalan parasite *Polymorphus minutus* is a manipulator of its intermediate host *Gammarus roeseli*, which favours its transmission to the final host, a water bird. In contaminated environments, *G. roeseli* have to cope with two stresses, i.e. *P. minutus* infection and pollutants. As *P. minutus* survival relies on its host's survival, we investigated the influence of *P. minutus* on the antitoxic defence capacities and the energy reserves of *G. roeseli* females after cadmium exposure. In parallel, malondialdehyde, a toxic effect biomarker, was measured in *G. roeseli* females and in *P. minutus*. The results revealed that infected females displayed higher cell damage than uninfected ones, despite an apparent increase in reduced glutathione and metallothionein production. In fact, the increase of these antitoxic systems could be counterbalanced by carotenoid intake by the parasite, so that the overall defence system seemed less efficient in infected females than in uninfected ones. In addition, we demonstrated that cadmium induced cell damage in *P. minutus*, probably linked with cadmium accumulation in the parasite. Altogether, we observed a paradoxical pattern of responses suggesting that *P. minutus* increases cadmium toxicity in *G. roeseli* females although (i) it tends to increase several host antitoxic defence capacities and (ii) it bears part of the pollutant, as reflected by cell damage in the parasite.

## Introduction

Over the last decade the study of parasitism in ecotoxicology has arisen increasing interest, and numerous studies have already underlined the influence of parasites on infected host physiology in contaminated environments (bivalves [1], [2]; arthropods [3]–[5]; fish [6]). Parasites need energy for their own development; thus they affect the host energy allocation [7], which could weaken host detoxification processes. Parasites can disrupt the antitoxic defences of their hosts [5], [8] as well as their immune system [9]. Conversely, some studies have described that parasites can help their host to cope with pollutants, for example by accumulating heavy metals in their own tissues [10], [11] or by increasing antioxidant enzyme activities [12]. Most of these studies were conducted in freshwater environments.

Gammarid amphipods are increasingly used in ecotoxicological studies in both freshwater and marine environments, especially due to their important role in the trophic chain [13]. They can be infected by several parasites such as nematodes, trematodes [14], [15], microsporidia [5], [16]–[18] or acanthocephalans [19]–[22].

Acanthocephalans are the most widely studied parasites in gammarids. Their complex life cycle includes an intermediate host (an arthropod) to grow and a final host (a vertebrate) to mature and reproduce. Acanthocephalan parasites at the cystacanth stage are known to alter the phenotype of their intermediate host by a behavioural manipulation, in a way that makes it more prone to predation and thus favours their transmission to the final vertebrate host [23], [24]. Although acanthocephalan parasites need their gammarid hosts in order to develop before being transmitted to the final host, in polluted environments, they may represent an additional burden for gammarids. Indeed, in contaminated ecosystems, gammarids are faced with two different stresses: the presence of the parasite and the toxicity of pollutants. In a previous study, we demonstrated that the acanthocephalan *Polymorphus minutus* influenced the cadmium resistance of its intermediate host *Gammarus roeseli*, by increasing or decreasing the cadmium LC_50_ (lethal concentration that caused the death of 50% of individuals) after 96 hrs exposure in males and females, respectively [11]. In that study, cadmium accumulation in *P. minutus* as well as a lower cadmium bioaccumulation in infected gammarids than in uninfected ones were observed. However, up to now, only few studies have investigated the effect of an acanthocephalan parasite on the host antitoxic defence capacities, which could explain the difference in resistance between uninfected and infected gammarids [8], [25], [26]. Hence, in a previous study, we described a reduction of the reduced glutathione concentration, a scavenger of metallic and organic xenobiotics, as well as the activity of glutathione synthesis in *P. minutus*-infected *G. roeseli* in the absence of environmental stress [25]. It was also highlighted that the cystacanth stage of *P. minutus* prevents the synthesis of heat shock protein 70 in *G. roeseli*, subjected to thermal disturbance or palladium exposure [8]. In addition, a weakening of two major parameters of crustacean immunity, i.e. the prophenoloxidase system and haemocyte concentration, have been shown in *Gammarus pulex* infected by one of the three following acanthocephalan parasites: *Pomphorhynchus laevis*, *P. tereticollis* and *P. minutus* [26].

Accordingly, in this study we hypothesized that in a contamination context, the parasite could influence host antitoxic defences, to provide host with a better defence capacity and therefore lead to increased host survival. Host survival is believed to favour parasite survival and consequently its transmission. We tested our hypothesis by determining, in controlled laboratory experiments, the effect of a cadmium stress on the antitoxic defence capacities and energy reserves in uninfected and *P. minutus*-infected *G. roeseli*. Antitoxic defences were assayed by measuring concentrations of reduced glutathione (GSH), a tripeptide that plays an essential role in the detoxification system by scavenging organic or metallic xenobiotics, responsible of oxidative stress (i.e. production of reactive oxygen species), thanks to its thiol group [27]. In parallel, the activity of γ-glutamylcysteine ligase (GCL, EC 6.3.2.2), the limiting enzyme of the *de novo* glutathione synthesis, was assayed. Concentrations of metallothioneins (MT), which are involved in binding metal compounds thanks to the thiol groups of cysteine residues and contribute to protecting tissues against oxidative damage [28], [29], were measured. Their induction was related to metal exposure in many monitoring studies [30], [31]. Carotenoids, which are involved in reproduction [32] and in antioxidant defences [33], were also measured; as well as levels of malondialdehyde (MDA), product of the lipid membrane degradation (i.e. lipoperoxidation) which reflects cell damage, and is thus considered as a biomarker of toxic effect. Moreover, energy reserves were assessed by measuring total lipid and glycogen contents. The levels of glycogen are representative of the energy available for current activities [34] whereas lipids are used during starvation or reproduction periods [35]. Finally, as acanthocephalan parasites contained carotenoïds [36], carotenoïd concentrations were measured in *P. minutus*, in parallel to lipid contents and malondialdehyde levels.

## Materials and Methods

### Sampling collection, maintenance and cadmium exposure

Due to the lack of infected males, this study was carried out only in *G. roeseli* females. Uninfected and *P. minutus*-infected non-ovigerous *G. roeseli* females were collected in April 2011 with a pond net in the French Nied River (Rémilly, North-eastern France, 49°00′N and 6°23′E), where cadmium concentrations were less than 0.2 µg.L^−1^(LADROME laboratory, Valence, France). Probably due to the fact that *P. minutus* castrate *G. roeseli* females, no infected females were found in precopulatory state (personal observation); thus, only females which were not mated to males were collected. Females were sorted out on the spot by observing gnathopods, which are smaller in females than in males. In addition, *P. minutus* cystacanths were easily identified in living individuals: they appeared as intense orange dots through the cuticle. The animals were transferred to the laboratory in large containers filled with river water, acclimated 5 days at 15°C in an Elendt M4 modified solution [37], and fed *ad libitum* with alder leaves. The Elendt M4 solution was modified due to the fact that no EDTA was added to avoid cadmium chelation during exposure.

Test solutions were prepared using Elendt M4 modified solution with CdCl_2_ added to obtain two cadmium exposure concentrations: 2 and 8 µg Cd.L^−1^. Controls consisted of Elendt M4-modified solution only. Cadmium concentrations were defined (i) from the LC_50_ (lethal concentration for 50% of individuals) within 96 hrs, which were 107 and 35 µg Cd.L^−1^ for uninfected and *P. minutus*-infected females, respectively [5]; and (ii) from the maximum admissible cadmium concentration in drinking water, which is 5 µg.L^−1^ (CD 98/83/EC, 1998). For each condition, two replicates of 50 females were exposed, to the different conditions at 15°C for 96 hrs, in a 1.5 L aquarium previously saturated with the corresponding cadmium solutions for 5 days. During exposure, animals were not fed. At the end of the exposure, cadmium concentration were measured and averaged 1.97±0.04 µg Cd/L and 7.96±0.05 µg Cd/L.

As *G. roeseli* females can be infected with microsporidia parasites, and to avoid co-infected females, the presence of microsporidia was investigated by PCR-RFLP [5], which allowed us to keep females infected by *P. minutus* for biomarker analyses. In *P. minutus* infected individuals, cystacanths were removed and stored individually at −80°C awaiting marker analyses. Similarly, gammarid bodies were individually frozen in liquid nitrogen and stored at −80°C awaiting biomarker analyses.

### *G. roeseli* biomarker assays

Assaying antitoxic defences is impossible to perform on individual gammarid, so a minimum number of six gammarids was necessary to get enough tissues to analyse all the parameters. Therefore, for each exposure condition, 6 replicates of 6 individual females with the same parasitic status (i.e. absence/presence of *P. minutus*) were made to measure energy reserves and antitoxic defences as described below. Two different conditions were established: (i) uninfected females and (ii) *P. minutus*-infected females.

#### Sample preparation

Each pool was homogenized with a Potter Elvejhem manual tissue grinder in 50 mM phosphate buffer KH_2_PO_4_/K_2_HPO_4_ (pH 7.6) supplemented with 1 mM phenylmethylsulphonylfluoride (PMSF) and 1 mM L-serine-borate mixture as proteases inhibitors, and 5 mM phenylglyoxal as a γ-glutamyl transpeptidase inhibitor. The homogenization buffer was adjusted to a volume two-fold the wet weight of the sample pool (e.g. 200 µL of homogenization buffer for 100 mg of wet weight tissue). The total homogenate was divided into seven parts to measure the different parameters. For each replicate, two independent measures were made for each biomarker.

#### Energy reserves

The measurement of total lipid and glycogen contents was adapted from Plaistow et al. [7]. A volume of 20 µL of 2% sodium sulphate (w/v) and 540 µL of chloroform/methanol 1∶2 (v/v) were added to 40 µL of the total homogenate. After 1 hr on ice, the samples were centrifuged at 3,000× g for 5 min at 4°C. The resulting supernatant and the pellet were used to determine the lipid and glycogen contents, respectively.

A volume of 100 µL of supernatant was transferred into culture tubes and placed in a dry bath at 95°C to evaporate the solvent. Then, 200 µL of 95% sulphuric acid were added in each tube and left for 10 min. The different tubes were removed and cooled on ice, and then 4.8 mL of phosphovanillin reagent were added. After a 10-min reaction, the optical density was measured at 535 nm. Commercial cholesterol was used as a standard and total lipid contents were expressed in mg.mL^−1^.

The pellets were dissolved in 400 µL of deionised water, 100 µL of sample were placed into culture tubes and 4.9 mL of Anthrone reagent were added. The mixture was placed in a dry bath at 95°C for 17 min and then cooled on ice. Optical density was measured at 625 nm. Glucose was used as a standard and concentrations were expressed in µg.mg^−1^ tissue.

The total protein content of each sample was quantified according to Bradford [38] with bovine serum albumin (BSA) as a standard. Results were expressed in mg.mL^−1^.

### Antitoxic defence capacities

#### Reduced glutathione concentration and γ-glutamylcysteine ligase activity

Reduced glutathione (GSH) concentrations were assessed by High-Pressure Liquid Chromatography (HPLC) separation adapted from Leroy et al. [39]. The proteins contained in 40 µL of the total homogenate were precipitated with 10% perchloric acid (v/v). After a 10-min centrifugation at 20,000× g and 4°C, the resulting supernatant was diluted 40-fold in 0.1 M HCl. Commercial reduced glutathione diluted in 0.1 M HCl was used as a standard and reduced glutathione concentrations were expressed in nmol GSH.mg^−1^ protein.

The γ-glutamylcysteine ligase (GCL) activity was assayed using an HPLC method adapted from Parmentier et al. [40]. Measurements were carried out on the S12000 fraction obtained after centrifuging 40 µL of the total homogenate for 15 min at 500× *g* and then centrifuging the resulting supernatant at 12,000× g and 4°C for 30 min. The resulting S12000 supernatant was diluted 20-fold in homogenization buffer and 40 µL of this diluted solution were added to 112 µL of incubation cocktail (0.5 M Tris HCl, 200 mM MgCl_2_ 6H_2_O, 500 mM KCl, 45 mM glutamic acid, 90 mM cystein, 1 mM DTT, 90 mM ATP, 0.5 mM phenylglyoxal, pH 8.25) to initiate the reaction. The mixture was incubated for 20 min at 25°C in a water bath and the reaction was stopped by a four-fold dilution with 0.1 M HCl. Commercial glutamylcysteine (GC) solution prepared in 0.1 M HCl was used as a standard and GCL activity was expressed in nmol GC.min^−1^.mg^−1^protein.

### Metallothionein assay

Metallothionein (MT) concentrations were determined with an HPLC method adapted from Alhama et al. [41]. A volume of 40 µL of the total homogenate was centrifuged at 3,500× g for 10 min. Then, the resulting supernatant was centrifuged at 22,000× g for 30 min and 4°C to obtain the S22000 fraction. A ten-fold dilution of the S22000 fraction was prepared in 100 mM Tris buffer (pH 9.5) supplemented with 1 mM DTT and 100 mM PMSF as a protease inhibitor. To reduce and denature the protein, 125 µL of diluted sample were added to 108 µL of incubation cocktail (230 mM Tris pH 9.5, 300 mM DTT, 100 mM EDTA and 10% sodium dodecyl sulfate) and placed in a water bath at 70°C for 20 min. Then, the incubation mixture was supplemented with 17 µL of 180 mM monobromobimane (mBBr) and incubated in the dark at room temperature for 15 min, to mark metallothioneins. Commercial rabbit-liver metallothionein I solution prepared in 230 mM Tris, pH 9.5, was used as a standard and metallothionein concentrations were expressed in nmol MT.mg^−1^ protein.

### Carotenoïd concentration

Carotenoid concentrations were measured by a spectrophotometry method adapted from Rauque and Semenas [42]. A volume of 40 µL of the total homogenate was diluted in 450 µL of 96% ethanol and kept 6 hrs in the dark at 4°C, before being centrifuged 10 min at 3,500× g. The optical density of the resulting supernatant was measured at 422, 448 and 476 nm, corresponding to the three major absorbance peaks observed in the absorption spectrum of *G. roeseli* (data not shown). A commercial carotene mixture (Sigma-Aldrich, France) was used as a standard. Carotenoid concentrations were expressed in ng carotenoids.mg^−1^ lipid.

### Toxic effect biomarker

Malondialdehyde (MDA) levels were determined using an HPLC method adapted from Behrens and Madère with UV detection at 267 nm [43]. A volume of 70 µL of the total homogenate was diluted four-fold in 95% ethanol (HPLC grade) and cooled on ice for 1.5 hrs to deproteinize it. The mixture was then centrifuged at 18,000× g for 30 min at 4°C. A volume of 100 µL of the resulting supernatant was injected into the HPLC separation system. Malondialdehyde levels were expressed in ng MDA.mg^−1^ lipid.

### *P. minutus* biomarker assays

In *P. minutus*, biomarkers were measured on 4 replicates of 9 *P. minutus* cystacanths each for each exposure condition. Each replicate was crushed in 350 µL of 96% ethanol and kept 1.5 hrs in ice. The mixture was then centrifuged at 3,000× g for 5 min at 4°C, and the resulting supernatant was divided into three parts to measure lipid contents, carotenoïd concentrations and malondialdehyde levels.

### Lipid contents

A volume of 60 µL of the resulting supernatant was two-fold diluted in chloroform-methanol 1∶2 (v/v). Then, 100 µL of this diluted solution were used to measure lipid contents as described above.

### Carotenoïd assays

A volume of 150 µL of the resulting supernatant was added to 300 µL of 95% ethanol, mixed and kept 6 hrs in the dark at 4°C. Carotenoïds were then estimated as described above.

### Malondialdehyde levels

A volume of 100 µL of the resulting supernatant was directly injected into the HPLC system, to measure malondialdehyde levels as described above.

### Statistical analyses

All data met normality and homogeneity of variance assumptions (Shapiro and Bartlett tests, *p*>0.05). Our data were analysed by using a multivariate analysis of variance (MANOVA, Pillai's trace) with respect to “infection status” and “cadmium exposure” as fixed factors, to test global effect of parasites and cadmium on all biomarkers. Since the MANOVA test was significant, each biomarker was then analysed using ANOVA tests, to test the effect of the infection status, cadmium exposure and their interaction. Then, TukeyHSD post-hoc tests were used to describe significant differences. All tests were performed with a 5% type-I error risk, using R 2.9.0 Software.

## Results

Global MANOVA analysis (Table 1) and ANOVAs tests (Table 2) revealed an effect of infection status, cadmium exposure, and their interactions on biomarker levels. Indeed, infection status had an effect on lipids and glycogen contents as well as on carotenoid and malondialdehyde levels. Cadmium exposure influenced all measured biomarkers. Finally, the interaction between infection status and cadmium exposure had influence all biomarkers, except metallothioneins concentration. As interaction between infection status and cadmium were significant for all biomarkers (almost significant for metallothionein), we described below biomarker results according to this interaction.

**Table 1:** Multivariate analyses of variance (Pillai's trace) investigating variations in energy reserves (lipid, glycogen) and defence capacity (GSH, GCL, MT, Carotenoid, MDA) of *Gammarus roeseli*, as a function of infection by acanthocephalan parasites and cadmium exposure.

Source of variation	num d.f.^a^, den d.f.^b^	F	*p*-value
**Infection status**	7, 24	7.14	<0.001
**Cadmium exposure**	14, 50	19.38	<0.001
**Infection : Cadmium**	14, 50	15.73	<0.001

a Numerator degrees of freedom.

b Denominator degrees of freedom

**Table 2:** Univariate analyses of variance (ANOVA) investigating variations in energy reserves (lipid, and glycogen), defence capacity (GSH, GCL, MT, carotenoid), and toxicity biomarker (MDA), in *Gammarus roeseli*, according to infection by *P. minutus* and cadmium exposure.

	Source of variation	Df	Mean square	F	*p*-value
**Lipid**	Whole model	5	-	27.23	**<0.001**
	Infection status	1	0.84	5.69	**0.024**
	Cadmium exposure	2	9.08	61.48	**<0.001**
	Infection : Cadmium	2	0.55	3.73	**0.035**
	Residuals	30			
**Glycogen**	Whole model	5	-	84.33	**<0.001**
	Infection status	1	0.29	11.65	**0.002**
	Cadmium exposure	2	4.39	178.61	**<0.001**
	Infection : Cadmium	2	0.65	26.4	**<0.001**
	Residuals	30			
**GSH**	Whole model	5	-	53.84	**<0.001**
	Infection status	1	0.029	0.401	0.53
	Cadmium exposure	2	7.39	100.8	**<0.001**
	Infection : Cadmium	2	2.46	33.6	**<0.001**
	Residuals	30			
**GCL**	Whole model	5	-	21.40	**<0.001**
	Infection status	1	0.002	0.56	0.49
	Cadmium exposure	2	0.144	46.84	**<0.001**
	Infection : Cadmium	2	0.02	6.38	**0.005**
	Residuals	30			
**MT**	Whole model	5	-	33.62	**<0.001**
	Infection status	1	0.063	0.616	0.439
	Cadmium exposure	2	8.3	80.71	**<0.001**
	Infection : Cadmium	2	0.31	3.08	0.063
	Residuals	30			
**Carotenoid**	Whole model	5	-	34.45	**<0.001**
	Infection status	1	9.11	7.33	**0.011**
	Cadmium exposure	2	97.47	78.39	**<0.001**
	Infection : Cadmium	2	5.07	4.07	**0.02**
	Residuals	30			
**MDA**	Whole model	5	-	24.92	**<0.001**
	Infection status	1	1.94	11.31	**<0.001**
	Cadmium exposure	2	7.75	45.25	**<0.001**
	Infection : Cadmium	2	1.95	11.39	**<0.001**
	Residuals	30			

The significant differences are indicated in bold.

### Effect of *P. minutus* without cadmium exposure

**Figure 1:**
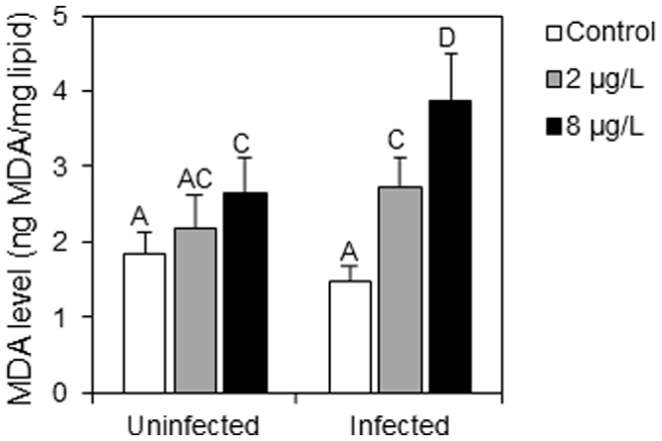
Malondialdehyde levels (ng.mg^−1^ lipids) in uninfected and *P. minutus*-infected *G. roeseli* females exposed at two cadmium concentrations (2 and 8 µg Cd.L^−1^) for 96 hrs. Error bar represent mean ± SE. Different letters above the bars indicate significantly different values (Tukey's HSD test, *p*-values<0.05).

**Figure 2:**
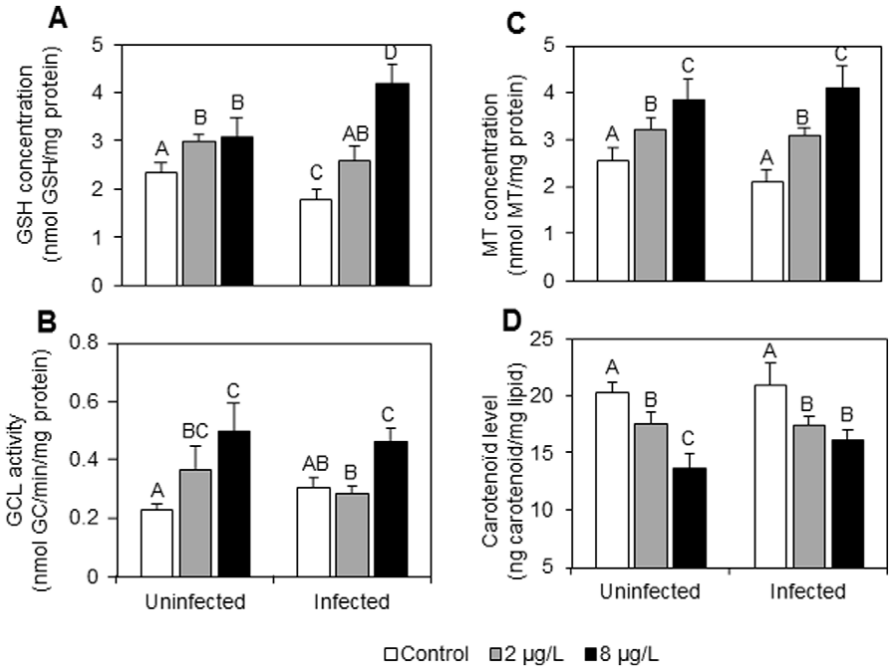
Antitoxic defense responses in uninfected and *P. minutus*-infected *G. roeseli* females exposed at two cadmium concentrations (2 and 8 µg Cd.L^−1^) for 96 hrs. A: GSH concentrations (nmol.mg^−1^ protein). B: GCL activity (nmol GC.min^−1^.mg^−1^ protein). C: MT concentrations (nmol.mg^−1^ protein). D: carotenoïd levels (ng.mg^−1^ lipids). Error bar represent mean ± SE. Different letters above the bars indicate significantly different values (Tukey's HSD test, *p*-values<0.05).

**Figure 3:**
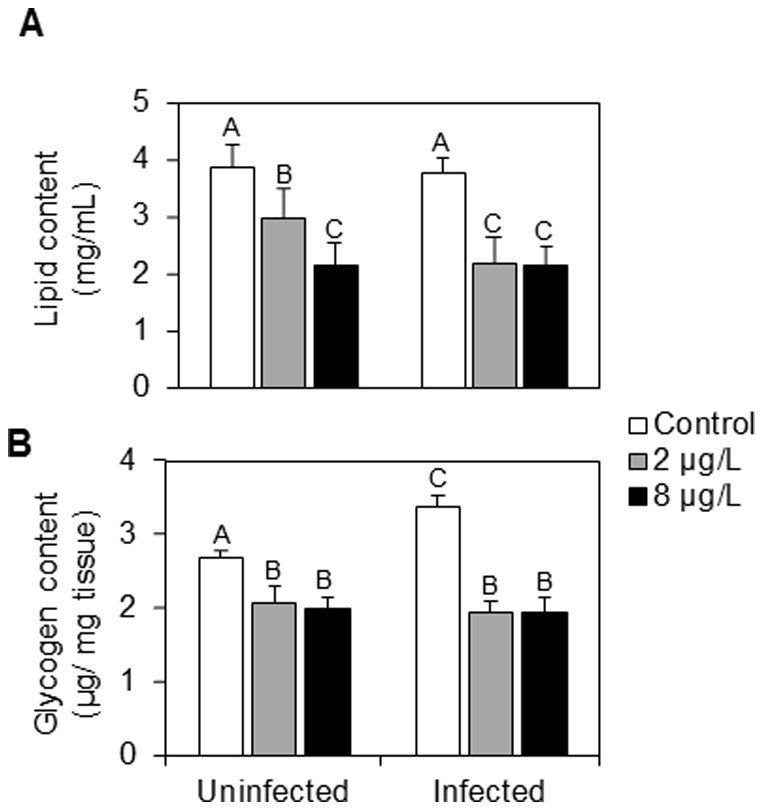
Energy reserve levels in uninfected and *P. minutus*-infected *G. roeseli* females exposed at two cadmium concentrations (2 and 8 µg Cd.L^−1^) for 96 hrs. A: total lipid content (mg.mL^−1^). B: glycogen content (µg.mg^−1^ tissue). Error bar represent mean ± SE. Different letters above the bars indicate significantly different values (Tukey's HSD test, *p*-values<0.05).

As shown in the figures and the post-hoc tests, infection by the acanthocephalan parasite *P. minutus* have influenced *G. roeseli* energy reserves, antitoxic defences and toxicity biomarkers (see white bars in Figure 1, Figure 2 and Figure 3). Indeed, malondialdehyde (MDA) level, lipid content and metallothionein (MT) concentration were unchanged but have a tendency to be lower in infected females than in uninfected ones (Figure 1, Figure 2C, Figure 3A). Reduced glutathione (GSH) concentrations were lower (Figure 2A) whereas γ-glutamylcysteine ligase (GCL) activity has a tendency to be slightly higher in infected females as compared to uninfected ones (Figure 2B). Conversely, carotenoïd concentrations and lipid contents remained unchanged in the presence of *P. minutus* (Figure 2D, Figure 3A), while glycogen contents were higher in *P. minutus*-infected females than in uninfected ones (Figure 3B).

### Effects of *P. minutus* on *G. roeseli* exposed to cadmium

#### Toxicity biomarker

Malondialdehyde (MDA) levels increased in uninfected and in *P. minutus*-infected females exposed to cadmium as compared to the respective controls (Figure 1). In uninfected females, MDA levels were 1.5-fold higher at 8 µg Cd.L^−1^, while only a limited increase was observed at 2 µg Cd.L^−1^. In *P. minutus*-infected females, MDA levels increased according to a dose-response relationship. Indeed, they were 1.8- and 2.6-fold higher in infected females exposed to 2 and 8 µg Cd.L^−1^, respectively, as compared to infected controls. Infection by *P. minutus* was therefore linked to higher MDA levels in the case of cadmium exposure, especially at 8 µg Cd.L^−1^.

### Defence capacities

Reduced glutathione (GSH) concentrations increased in both infection statuses (Figure 2A). In uninfected females, GSH concentrations significantly increased with cadmium exposure, but no significant difference was observed between the two cadmium concentrations. However, a dose-response relationship was observed in infected females. GSH concentrations were 1.3 and 2-fold higher when infected gammarids were exposed to 2 and 8 µg Cd.L^−1^, respectively, as compared to unexposed ones.

The γ-glutamylcysteine ligase (GCL) responses were also influenced by the presence of *P. minutus* (Figure 2B). Indeed, in uninfected females, GCL activity increased according to a dose-response relationship. The activity was 1.7- and 2.3-fold higher with the lowest and the highest cadmium concentrations, respectively. In *P. minutus*-infected females, a significant increase was only observed at 8 µg Cd.L^−1^, when GCL activity increased 1.6-fold.

Metallothionein (MT) concentrations in exposed gammarids did not significantly differ between uninfected and infected females (Figure 2C). Whatever the infection status, a dose-response relationship was observed. In uninfected females, MT concentrations were 1.2- and 1.6-fold higher with 2 and 8 µg Cd.L^−1^, respectively; while in infected females, they were 1.5- and 2-fold higher in the same conditions.

Carotenoïd levels decreased in uninfected females according to a dose-response relationship (Figure 2D). In fact, at 2 µg Cd.L^−1^, carotenoïd concentration was 1.2-fold lower, while at 8 µg Cd.L^−1^, it was 1.5-fold lower. In infected females, whatever the cadmium exposure, carotenoïd concentrations were 1.2-fold lower.

### Energy reserves

Energy reserves were influenced by the presence of *P. minutus* (Figure 3). Uninfected females showed a significant decrease in total lipid contents whatever the cadmium exposure (Figure 3A). In fact, lipid contents were 1.3- and 1.9-fold lower at 2 and 8 µg Cd.L^−1^ respectively. In infected females, lipid contents were also lower but no significant difference was observed between the two cadmium concentrations, as total lipid contents were 1.8-fold lower at 2 and 8 µg Cd.L^−1^.

The glycogen contents of uninfected and *P. minutus*-infected females were decreased following the same pattern, whatever the cadmium exposure (Figure 3B). Indeed, at the two cadmium concentrations, glycogen contents were 1.3-fold lower in uninfected females, while they were 1.7-fold lower in infected ones.

### Effect of cadmium on *P. minutus* biomarkers

Global MANOVA analysis revealed an effect of cadmium exposure on biomarker levels (*p*-value<0.001). The ANOVAs tests were presented in table 3.

**Table 3:** Univariate analyses of variance (ANOVA) investigating variations in lipid content, carotenoid concentration and MDA levels in *P. minutus* according to cadmium exposure.

		Df	Mean square	F	*p*-value
**Lipid**	Cadmium	2	0.454	75.38	**<0.001**
	Residuals	9			
**Carotenoid**	Cadmium	2	5.25	22.42	**<0.001**
	Residuals	9			
**MDA**	Cadmium	2	0.001	109.44	**<0.001**
	Residuals	9			

**Figure 4:**
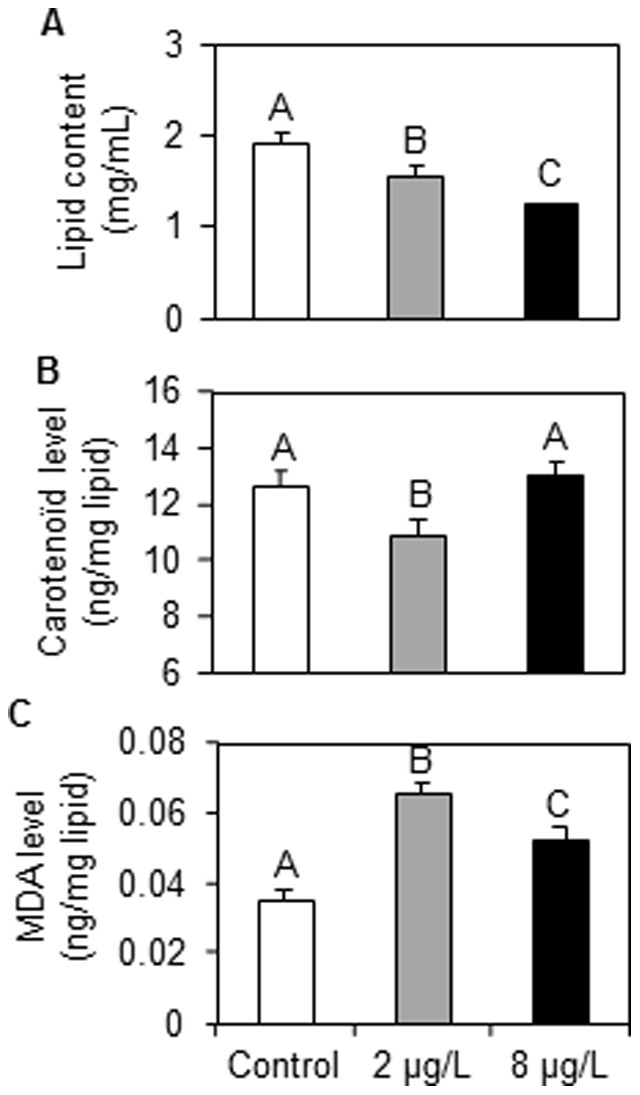
Lipid, carotenoid and malondialdehyde levels in *P. minutus* dissected from *G. roeseli* females exposed at 2 and 8 µg Cd.L^−1^ for 96 hrs. A: Total lipid contents (mg.mL^−1^). B: carotenoid (ng.mg^−1^ lipids). C: MDA (ng.mg^−1^ lipids). Error bar represent mean ± SE. Different letters above the bars indicate significantly different values (Tukey's HSD test, *p*-values<0.05).

Lipid contents, carotenoïd and MDA levels were also measured directly in *P. minutus*. The results are presented in figure 4.

Lipid contents in the parasite also decreased according to a dose-response relationship (Figure 4A). In fact, lipid contents were 1.3- and 1.6-fold lower at 2 and 8 µg Cd.L^−1^, respectively. In parallel, carotenoïd concentrations were measured and the results revealed a significant decrease only at 2 µg Cd.L^−1^ but not at 8 µg Cd.L^−1^ (Figure 4B).

MDA levels were 1.9- and 1.4-fold higher in *P. minutus* exposed at 2 and 8 µg Cd.L^−1^, respectively (Figure 4C).

## Discussion

This present work was carried out to investigate the influence of acanthocephalan cystacanth on the antitoxic defences and energy reserves of their intermediate host *G. roeseli* in an environmentally relevant cadmium exposure. Indeed, although macroparasites have already been shown to decrease significantly heavy metal accumulation in their hosts [8], [44], [45], the influence of these parasites on antitoxic responses had remained quite overlooked so far.

### *P. minutus* effects in the absence of cadmium stress

In the absence of cadmium stress (white bars in figures 1 to 3), *P. minutus* influenced *G. roeseli* biomarkers following different patterns. Malondialdehyde (MDA) levels were lower in infected-females than uninfected ones. This result is in accordance with one of our previous studies which showed a tendency towards lower MDA levels in *P. minutus*-infected gammarids [25]. This reflects lower cell damage, probably due to host protection by the parasite whose survival depends on its host's survival. However, this result is contradictory to the variations observed in defence capacities, especially reduced glutathione (GSH) and metallothionein (MT) concentrations which were lower in infected females than in uninfected ones, when gammarids were not exposed to stress. Indeed, weaker antitoxic defence capacities could allow us to predict that gammarid sensitivity should increase in contaminated environments. Other investigations have also shown lower MT and GSH concentrations in infected individuals [25], [44], [45], as well as lower immunity parameters (i.e. prophenoloxidase activity–[26]). Carotenoïd concentrations were not impacted by the presence of *P. minutus* although they are used by *P. minutus* for its own development [36], [46]. This result was not expected as (i) carotenoïds are synthesized neither by the parasite nor by the host, and (ii) the characteristic colour of the cystacanth stage is based on an accumulation of carotenoïds coming from its host.

*P. minutus* cystacanths influenced the energy reserves of their hosts by increasing glycogen contents and decreasing (or tending to decrease in this study) lipid contents. These results are in accordance with those obtained in one of our previous studies [25] and also with those of Plaistow et al. [7] or Cornet et al. [47] who have observed an increase of glycogen content and a tendency of decrease in lipids content, respectively. Lipid depletion could be explained by the fact that parasites draw out energy for their own development from their host [48]. However, glycogen increases could be linked to gammarid immobility as a consequence of parasite infection that makes the host-parasite system more vulnerable to final host predation [20], [49].

### Influence of *P. minutus* on *G. roeseli* antitoxic responses under cadmium stress

Although a possible protective effect of *P. minutus* on *G. roeseli* was observed in the absence of stress, the results obtained under cadmium stress highlighted higher cell damage in infected females as compared to uninfected ones. Indeed, whatever the infection status, cadmium exposure induced lipoperoxidation (i.e. increased malondialdehyde levels), but cell damage was higher in the presence of the parasite. This result is in agreement with those obtained when *G. roeseli* females were infected by microsporidia parasites [5] and could be explained by a disruption of the antitoxic defence responses.

Thus, the antitoxic responses of infected females were different from those of uninfected ones. Metallothionein concentrations, whose synthesis is known to be induced by several metals including cadmium [50], tended to increase faster according to a dose-response relationship in *P. minutus*-infected females as compared to uninfected ones. We can hypothesize that *P. minutus* could influence the transcription of the metallothionein gene to provide higher concentrations in its host than in uninfected individuals and cope with cadmium stress by sequestering the metal. However, our result is in contradiction with those of Baudrimont et al. [51] who showed lower metallothionein concentrations in digenean-infected cockles exposed to cadmium as compared to uninfected ones, and of Paul-Pont et al. [52] who highlighted lower metallothionein concentrations in the bivalve *Ruditapes philippinarum* infected by the trematode *Himasthla elongate* and/or the bacterium *Vibrio tapetis* and exposed to cadmium. In these two studies, the results were explained by the fact that other efficient intracellular metal ligands such as glutathione could interfere with cadmium sequestration process [53]. Our results showed that glutathione concentrations also increased according to a dose-response relationship in infected females as compared to uninfected ones. The highest reduced glutathione concentration we measured was obtained with the highest Cd exposure in infected females. This increase observed in infected females could be linked to the increase in GCL activity at the highest cadmium concentration. In uninfected females exposed to the highest cadmium concentration, the relatively low GSH concentration combined with increased GCL activity could result from the fact that glutathione could be directly used as a metal scavenger as well as a substrate for antioxidant enzymes [54]. We can thus hypothesize that *P. minutus* could influence on the activity of these antioxidant enzymes, which could lead to less glutathione being used and thus a weaker decrease in GSH concentration.

The important increase in GSH and MT concentrations could also be linked to the fact that carotenoid concentration decreased less in infected females as compared to the uninfected ones, according to a dose-response relationship. Indeed, no difference was observed in carotenoïd concentrations between the lowest and the highest cadmium exposures in infected females. This could be explained by the fact that *P. minutus* needs carotenoïds for its own metabolism [46]; therefore, it could reduce carotenoïd use of *G. roeseli* by drawing them from its host, and hence maintain a sufficient stock for itself. Indeed, in gammarids, this carotenoïd stock cannot be completed because gammarids cannot synthesize carotenoïds and must get them from food [55]; however, in our study, gammarids were not fed during cadmium exposure.

All antitoxic defence capacities involve an energy cost for the organism; however, in infected females, energy could be reallocated by the parasite to its own metabolism [7]. Our results highlighted a strong decreased in lipid and glycogen contents starting from the lowest cadmium exposure in infected females, whereas in uninfected females, these parameters decrease according to a dose-response relationship. On the one hand, these decreases could be explained by an energy mobilization by *G. roeseli* to cope with cadmium stress as already observed in several ecotoxicological investigations [5], [54], [56], [57]. However, on the other hand, the strong decreases observed in infected females could also be linked to the fact that the parasite diverts energy from its host not only for its own development, but also for its own defence system. In a previous study, we observed that *P. minutus* located within the *G. roeseli* hemocoel accumulated cadmium [11], which could explain the higher cell damage measured in cadmium-exposed *P. minutus* than in controls. If the parasite accumulates cadmium, we hypothesize that it can also detoxify it. The carotenoïd depletions observed in this work in *P. minutus* at the lowest cadmium exposure could support this hypothesis as they are antioxidant compounds [33], possibly linked to a detoxification process. As in *G. roeseli* females, total lipid contents in *P. minutus* decreased in a dose-response relationship; this observation is consistent with a potential lipid mobilization to provide energy to detoxification processes. This hypothesis is supported by the fact that Sures and Radszuweit [8] described an induction of the Hsp70 defence protein in *P. minutus* exposed to thermal disturbance.

## Conclusion

This study highlights the influence of the *P. minutus* cystacanth on the antitoxic defence capacities and the energy reserves of its intermediate host *G. roeseli* females in cadmium stress conditions. The results revealed that *P. minutus* could increase the use of some antitoxic defences (reduced glutathione and metallothionein), whereas others (carotenoïds) necessary for its own metabolism were maintained. However, the fact that *G. roeseli* females did not use all these antitoxic defences could explain the toxicity increase reflected by higher MDA levels. To our knowledge, this is the first study that shows a toxic effect of cadmium in *P. minutus* cystacanths; probably linked to cadmium absorption by the parasite [11]. Since our study was conducted only in females, it could be interesting to compare these results to the influence of *P. minutus* on biomarkers of *G. roeseli* males. Indeed, in our previous study [11], we demonstrated that *P. minutus*-infected males were more resistant to cadmium as compared to uninfected ones. Thus, it would not be surprising to observe differences in the parasite influence according to the host gender, since different antitoxic defences capacities were observed between uninfected males and females [53].
